# Support from start to finish—a collaborative primary medical program in rural Victoria, Australia

**DOI:** 10.3389/fmed.2025.1585017

**Published:** 2025-07-21

**Authors:** Will Harvey, Lachlan Van Schaik, Tamekha Develyn, Zahra Ali, Cathryn Hogarth, Julian Wright

**Affiliations:** ^1^Department of Rural Health, The University of Melbourne, Shepparton, VIC, Australia; ^2^Department of Rural Clinical Sciences, La Trobe Rural Health School, Wodonga, VIC, Australia

**Keywords:** distributed medical education, rural, Australia, rural medical education, medical workforce shortage, health workforce policy

## Abstract

Australia faces a persistent shortage of doctors in rural and regional areas, exacerbating health disparities between urban and rural communities. Traditional medical education models, which have been largely centralized in metropolitan areas, often result in rural-origin students needing to relocate to cities for training, thus disrupting community connections and reducing the likelihood of their return to rural practice. To address this challenge, the University of Melbourne and La Trobe University have collaborated to establish Victoria’s first end-to-end rural medical pathway, an innovative model that enables students to complete both their undergraduate [“Bachelor of Biomedical Science (Medical)”] and Doctor of Medicine (MD) entirely within regional and rural settings. This paper explores the implementation, practical considerations, and evaluation mechanisms of the end-to-end rural medical pathway, highlighting its place-based curriculum, and fully distributed medical education model. Although this program is yet to be evaluated, it is intended that by embedding students in primary care clinics and regional hospitals throughout their training, the program will foster long-term professional and personal ties to rural communities. This initiative represents a scalable and evidence-based model for addressing rural medical workforce shortages, offering insights that could inform national and international medical education policy.

## 1 Introduction

### 1.1 Context

Australia faces persistent challenges in providing equitable healthcare access to its rural and regional communities, which encompass approximately 28% of the population. These areas are characterized by vast geographical distances, smaller population densities, and limited healthcare infrastructure, all of which contribute to significant health disparities (1). Rural Australians experience poorer health outcomes, including higher rates of chronic diseases, mental health conditions, and preventable hospitalizations, compared to their urban counterparts (1, 2). Additionally, these communities face a higher burden of disease and mortality (1), further underscoring the need for targeted interventions. One way of identifying areas in need of such targeted interventions is by the use of the Modified Monash Model (MMM), which classifies rural and remote areas in Australia into seven categories based on factors such as population size, accessibility to services, and geographical remoteness (3).

Medical workforce maldistribution persists as one of the major challenges in providing equitable healthcare access in rural and regional communities. This challenge is compounded by the centralization of medical education in metropolitan areas, which often necessitates the relocation of rural students to urban centers for training. Such movement disrupts connections to rural communities, a factor known to reduce the likelihood of these individuals returning to practice in rural settings (4). Moreover, rural healthcare providers often encounter unique challenges, including professional isolation, limited access to resources, and broader scope of practice demands, which can deter potential rural doctors (5).

### 1.2 The problem

Despite significant government investment in initiatives aimed at bolstering the rural healthcare workforce, many communities remain underserved. Current national statistics reveal stark inequalities: while approximately 28% of Australians live in rural areas, only about 16% of doctors practice in these regions (1). This disparity is particularly pronounced in remote and very remote areas, classified as MMM6 and MMM7 under the Modified Monash Model, where healthcare access is severely limited (1).

The traditional undergraduate degree and/or Doctor of Medicine (MD) models of medical education, heavily concentrated in metropolitan centers, exacerbate these issues by creating barriers for students from rural backgrounds (6). Evidence suggests that students with rural origins are more likely to return to rural practice after graduation, but many face challenges in accessing medical training close to home (4, 7). Furthermore, the lack of continuity between most undergraduate degrees and MD programs disrupts the rural training pipeline, making it difficult for students to seamlessly progress through their medical education without relocating to urban centers. This fragmentation weakens the effectiveness of rural workforce initiatives and limits the number of doctors undertaking or remaining in rural practice (8–10).

### 1.3 The solution

**FIGURE 1:**
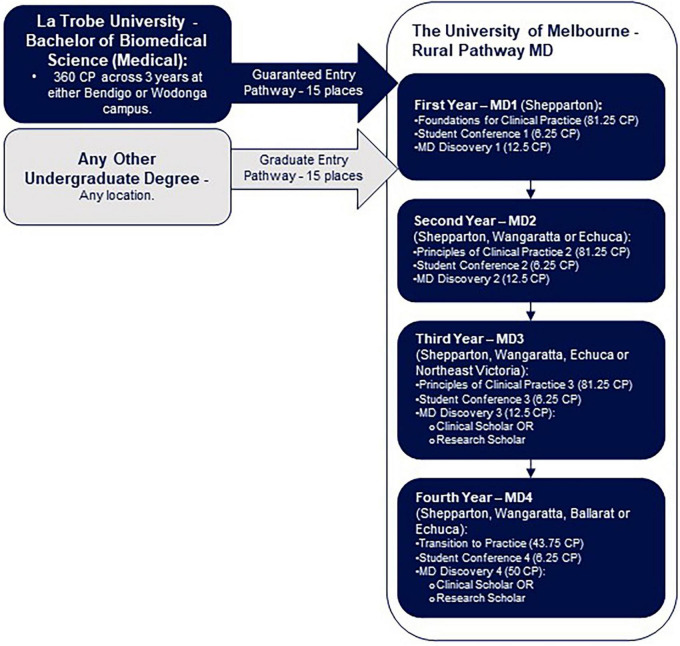
Visual representation of the end-to-end rural medical pathway, including possible locations of study. The Bachelor of Biomedical Science (Medical) at La Trobe University (Bendigo or Wodonga), including joint recruitment efforts with The University of Melbourne, forms the guaranteed entry pathway (15 places). Students may also enter the Rural Pathway MD via the graduate entry pathway, after completing any other undergraduate degree (15 places). Abbreviations: Credit points (CP); MD (Doctor of Medicine).

**FIGURE 2:**
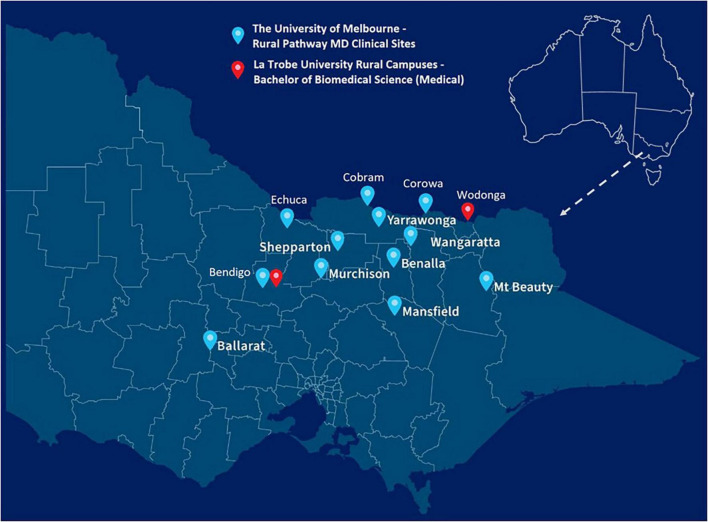
The end-to-end rural medical pathway—consisting of the Bachelor of Biomedical Science (Medical) at La Trobe University, and the Rural Pathway MD at The University of Melbourne—study locations across rural Victoria.

To address these systemic challenges, the University of Melbourne’s Department of Rural Health and La Trobe University Rural Health School have collaborated to establish Victoria’s first end-to-end rural medical pathway (Figure 1). This innovative program seeks to address both the shortage of rural healthcare professionals and the challenges faced by rural-origin students in accessing medical training. By leveraging the Victorian regional footprint of both institutions (Figure 2), this program provides a comprehensive training pathway that spans both undergraduate degree and Doctor of Medicine and eliminates the need for rural students to relocate to metropolitan areas for their medical training. This program also limits eligibility so that only students from regional and rural backgrounds can participate. By embedding regional and rural students in rural communities throughout their training, the program intends to foster long-term relationships with local healthcare providers and patients, strengthening commitment to rural practice (2, 4, 11).

The program’s structure emphasizes place-based education, integrating the training environments of primary care clinics, and regional hospitals. Students benefit from hands-on learning opportunities tailored to the specific needs of rural populations, including management of undifferentiated health problems, chronic disease care, and preventive health strategies. Moreover, the curriculum incorporates cultural competence training and interprofessional collaboration; essential skills for addressing the unique challenges of rural healthcare delivery.

Leveraging smaller cohort sizes, in comparison to the metropolitan cohort, is just one way the program ensures personalized attention and mentorship, enhancing medical students’ confidence in clinical, procedural, and community health skills. This approach aligns with findings from Ellaway and Bates (12), who emphasized the transformative potential of distributed medical education (DME) in fostering a culture of learning and resilience among medical trainees.

### 1.4 Objective

This paper aims to provide insights into the program’s implementation, practical considerations, and evaluation mechanisms. By showcasing the potential of this program as a scalable model, the paper also aims to inform national and international discussions on rural medical education and workforce planning. The program’s collaborative approach to spanning both undergraduate degree and Doctor of Medicine may serve as a blueprint for similar initiatives globally, addressing one of the most pressing challenges in rural healthcare.

## 2 Pedagogical frameworks, pedagogical principles, competencies/standards underlying the educational activity

The end-to-end rural medical pathway integrates elements from four pedagogical frameworks; competency-based education (CBE) (13, 14), distributed medical education (DME) (15), place-based learning (16), and social accountability (17–19). Adoption of these principles aligns the medical education, provided by the program, with the unique needs of rural healthcare settings.

### 2.1 Competency-based education

The end-to-end rural medical pathway is underpinned by a Competency-Based Education (CBE) framework, designed to ensure the systematic development of students’ knowledge, skills, and professional attitudes essential for effective medical practice (14, 20). The program’s intended learning outcomes (Supplementary Tables 1–3) and graduate attributes (Table 1) are aligned with the standards set by the Australian Medical Council (AMC), including competencies related to population health, health inequities, and the broader socio-economic and environmental determinants of health (21). While the AMC standards do not explicitly focus on rural and remote contexts, the program incorporates principles of distributed medical education (DME), place-based learning, and social accountability to address the specific health needs of rural communities.

**TABLE 1:** Rural pathway doctor of medicine—graduate attribute framework.

Attribute domain	Graduate attribute statement
Self—In building their relationship with self, students will develop:	1. An understanding of the principles of empathy, compassion, honesty, integrity, altruism, resilience and lifelong curiosity; the ability to demonstrate them and a recognition of their importance in health care
	2. An understanding of the principles of reflective practice, the ability to apply them, and a recognition of their importance in health care
	3. An understanding of the principles of self-awareness, the ability to recognize when clinical problems exceed their knowledge and skill, and a willingness to seek help
	4. The ability to identify and address their own learning needs
	5. The ability to respond constructively to appraisal, performance review or assessment
	6. The ability to manage uncertainty
	7. The ability to apply effective time management and organizational skills
	8. The ability to recognize and manage emotion in themselves and others
	9. The ability to maintain their own physical, emotional, social and spiritual health and a recognition of the importance of professional support in this process
	10. A recognition of their own personal, spiritual, cultural or religious beliefs and an awareness that these beliefs must not prevent the provision of adequate and appropriate care to the patient
Knowledge—In building their relationship with knowledge, students will develop:	1. An understanding of the scientific method relevant to biological, behavioral and social science
	2. An understanding of research methods and their applications
	3. An understanding of normal structure, function and development of the human body and mind at all stages of life
	4. An understanding of the molecular, biochemical and cellular mechanisms that are important in maintaining the body’s homeostasis
	5. An understanding of normal life processes including conception, development, birth, aging and death
	6. An understanding of the factors that might disturb normal structure, function and development
	7. An understanding of the etiology, pathology, symptoms and signs, natural history and prognosis of important physical and mental illnesses in all stages of life
	8. An understanding of the management (pharmacological, physical, nutritional, behavioral and psychological) of important medical conditions
	9. the ability to access new knowledge from all sources, to analyze and interpret it in a critical manner, and to apply it appropriately to their provision of health care
	10. The ability to learn from patients, health professionals and the community in a broad range of settings
	11. An appreciation of the responsibility to contribute toward the generation of new knowledge
Patients—In building their relationship with patients, students will develop:	1. An understanding of and respect for the rights of patients including patient choice, dignity and privacy
	2. The ability to communicate with patients from diverse backgrounds including the ability to listen to, respond to, inform and understand the patient’s perspective
	3. The ability to advocate appropriately on behalf of the patient
	4. An understanding of factors affecting human relationships and the psychological, cultural and spiritual wellbeing of patients
	5. An understanding of principles of rehabilitation in the amelioration of suffering from acute or chronic disability
	6. An understanding of the principles of the care of the dying and a commitment to ease pain and suffering in all patients
	7. An understanding of chronic illness and disability and its impact on the patient, their carers and communities
	8. The ability to construct with the patient an accurate, thorough, organized, medical history and to perform an accurate physical and mental state examination
	9. The ability to integrate and interpret clinical findings and apply rigorous reasoning to arrive at an appropriate diagnosis or differential diagnosis
	10. The ability to recognize serious illness
	11. The ability to select and interpret the most appropriate and cost-effective diagnostic procedures
	12. The ability to formulate an evidence-based and cost-effective management plan in collaboration with the patient
	13. The ability to perform relevant medical procedures effectively and safely, with due regard for the patient’s comfort including important emergency and life-saving procedures
	14. A recognition that it is not always in the interests of the patient to do everything that is technically possible to make a precise diagnosis or to attempt to modify the course of an illness
Medical Profession—In building their relationship with the medical profession, students will develop:	1. An understanding of the continuum of medical training and the diverse roles and expertise of doctors
	2. An understanding of the potential conflicts of interest that may confront doctors
	3. An understanding of and ability to apply the principles of ethics in the provision of health care and research.
	4. An understanding of organizational governance, the ability to be an active participant in professional organizations, and an appreciation of the benefits of this participation
	5. An understanding of the principles of mentorship and the ability to apply them with colleagues
	6. The ability to give effective feedback to colleagues in order to help them improve their performance
	7. An understanding of educational theory and practice and the ability to teach
	8. An appreciation of the responsibility to maintain standards of medical practice at the highest level throughout a professional career
Systems of Health Care—In building their relationship with systems of health care, students will develop:	1. An understanding of the roles, responsibilities and expertise of all health professionals, and how they work in teams to deliver health care
	2. A respect for the roles and expertise of other health care professionals and the ability to communicate effectively with them
	3. An understanding of the principles of teamwork and the ability to work effectively in a team, including as a leader
	4. An appreciation of the responsibility to contribute to the education of all health professionals
	5. An understanding of the principles of quality and safety in health care systems
	6. The ability to work effectively as a doctor within a quality and safety framework including the ability to recognize, respond to and learn from adverse events and medical errors
	7. An understanding of the principles of effective record keeping and the ability to maintain high quality medical records
	8. An understanding of the principles of continuity and coordination of health care
	9. An understanding of the structure of the Australian health care system and health care systems globally
	10. An understanding of the principles of efficient and equitable allocation and use of finite resources in health care systems, locally and globally
	11. An understanding of the role of political systems in shaping health care systems locally, nationally and internationally
Society—In building their relationship with society, students will develop:	In building their relationship with society, students will develop:
	1. An understanding of the interactions between humans and their social and physical environment
	2. An understanding of the determinants of a well society and the economic, political, psychological, social and cultural factors that contribute to the development and persistence of health and illness
	3. An understanding of the principles of health promotion including primary and secondary prevention
	4. An understanding of the health of Aboriginal and Torress Strait Islander peoples including their history, cultural development and the impact of colonization and the ongoing health disparities of Indigenous people in this country and globally
	5. An understanding of the burden of disease in differing populations and geographic locations
	6. An understanding of the differing requirements of health care systems in a culturally diverse society
	7. The ability to respect community values, including an appreciation of a diversity of backgrounds and cultural values
	8. An understanding of the principles of health literacy and a willingness and ability to contribute to the health education of the community
	9. The ability to consider local, regional, national and global ramifications of health care issues
	10. The ability and a willingness to contribute to the community
	11. A commitment to contribute to the resolution of health inequities locally and globally
	12. An understanding of the relationship between environmental issues and the health of local communities and society
	13. A commitment to practice medicine in an environmentally responsible way

### 2.2 Distributed medical education (DME): aligning education with rural needs

Distributed Medical Education provides a decentralized model of medical training, offering students prolonged and immersive exposure to rural healthcare environments (15). This model facilitates the development of generalist clinical competencies, enhances students’ understanding of rural health systems, and fosters interprofessional collaboration (15, 22). Through DME, students acquire essential skills in patient assessment, clinical reasoning, and team-based care—attributes particularly critical in rural settings, where medical practitioners frequently undertake a broad scope of practice within resource-constrained environments (23).

### 2.3 Place-based learning: connecting education with rural communities

Place-based learning grounds students’ education in the specific social, cultural, and healthcare contexts of the rural communities in which they train (16). This approach enables students to engage directly with health challenges commonly faced by rural populations, including geographic isolation, limited access to care, and the higher prevalence of chronic disease and mental health conditions. By developing a contextualized understanding of these issues, students are better prepared to deliver patient-centered care, build sustained therapeutic relationships, and tailor health interventions to community needs—directly supporting graduate competencies related to patient care and professional identity formation (16, 24).

### 2.4 Social accountability: ensuring education meets community needs

Social accountability is embedded throughout the Rural Pathway MD, ensuring that the program remains responsive to the health priorities of rural communities (17–19, 25). This commitment is operationalized through:

• Graduate tracking initiatives, which monitor career trajectories and the extent to which graduates contribute to rural health workforce capacity.
• Sustained community partnerships, which provide students with mentorship, clinical exposure, and curriculum relevance.
• An explicit focus on health equity, equipping students to identify and address social determinants of health and advocate for underserved populations.

Through the integration of social accountability, the program aims to produce graduates who not only demonstrate clinical competence but also serve as agents of change in rural health systems—fulfilling the graduate attributes aligned with societal contribution and equity in healthcare.

## 3 Learning environment (setting, students, faculty); learning objectives; pedagogical format

The University of Melbourne’s Rural Pathway Doctor of Medicine was developed through a collaboration between the University of Melbourne and La Trobe University as part of the Murray-Darling Medical Schools Network (MDMSN) (26). This program integrates what was an existing Bachelor of Science undergraduate degree, now a specific Bachelor of Biomedical Science (Medical) degree at La Trobe University, with the University of Melbourne’s accredited postgraduate Doctor of Medicine. A key aspect of program design was a collaborative consultation process to refine curriculum alignment, from undergraduate to Doctor of Medicine, facilitating a seamless transition between the two.

Given the separate accreditation requirements for the rural delivery of the Doctor of Medicine, specific accreditation processes were undertaken to ensure compliance with the Australian Medical Council, while maintaining program flexibility.

The Rural Pathway MD is specifically designed for domestic students from rural backgrounds who intend to complete their medical training entirely in rural settings and pursue future practice in regional or rural areas, thereby strengthening the non-metropolitan medical workforce. This program offers 30 bonded Commonwealth Supported Places (CSP) (27), equally distributed across two entry pathways. The bonded CSP funding model subsidizes students’ course fees in exchange for a commitment to work in a rural or regional area for a minimum of 3 years, within 18 years of graduation (27). Fifteen places are reserved for graduates of La Trobe University’s Bachelor of Biomedical Science (Medical) degree—based in Bendigo and Wodonga—selected at entry to this undergraduate degree and subject to satisfactory academic performance. The second potential entry point into the Rural Pathway MD and remaining 15 places are open to graduates from any discipline who can demonstrate rural origin and a strong commitment to rural practice. Notably, applicants for this graduate-entry pathway are not required to sit the Graduate Medical School Admission Test (GAMSAT) (28). Both the La Trobe undergraduate program and the University of Melbourne Rural Pathway MD use Multiple Mini Interviews (MMIs) as part of their selection processes, with participation from university staff and community representatives (29, 30). Eligibility and selection criteria are summarized in Table 2.

**TABLE 2:** End-to end rural medical pathway—eligibility and selection information.

		Selection criteria	Number of common wealth-supported places
End-to-End Rural Medical Pathway	Bachelor of Biomedical Science (Medical)—La Trobe University (Guaranteed Entry Pathway)	• Resided for at least 5 years consecutively or 10 years cumulatively in remoteness areas classified as Modified Monash Model (MMM) 2–7 (3). • Minimum Australian Tertiary Admission Rank (ATAR) of 80.00 (31). • Multiple Mini-Interviews (MMI) consisting of questions on advocacy, collaboration, critical thinking, empathy, ethical reasoning, motivation, and regional identification. • Applicants will be assigned to one of the following three rurality tiers before proceeding to the next stage of the selection process, prioritized according to tiers 1 through 3. • Tier 1: evidence of rural background in MMM2-7 postcodes part of either the Murray Primary Health Network or the Murrumbidgee Primary Health Network. • Tier 2: evidence of rural background in MMM2-7 in other areas of rural Victoria and rural NSW. • Tier 3: evidence of rural background in MMM2-7 in other areas of rural Australia.	15
	Rural Pathway MD—The University of Melbourne (Graduate Entry)	• Domestic student currently living in Australia. • Completed an undergraduate degree in any discipline within the past 10 years. • Resided for at least 5 years consecutively or 10 years cumulatively in remoteness areas classified as MMM2 to MMM7 (3). • Minimum GPA of 5. • No Graduate Medical School Admission Test (GAMSAT) requirement (28). • MMI consisting of questions on advocacy, collaboration, critical thinking, empathy, ethical reasoning, motivation, and regional identification.	15

### 3.1 Setting

The Bachelor of Biomedical Science (Medical) is a 3-year undergraduate degree. Offered at La Trobe University’s Wodonga and Bendigo campuses, students are able to express a preference for studying in Bendigo or Wodonga, with the result being either 7 or 8 students commencing at both campuses each year (Figure 2). Designed as a pre-medicine course (Figure 1), it utilizes subjects in several subdisciplines of biomedical science, including biochemistry, pharmacology, physiology and immunology, to provide a comprehensive understanding of the human body, its structure, and its functions (Table 3). The course intended learning outcomes are presented in Supplementary Table 1.

**TABLE 3:** Curriculum details for the end-to-end rural medical pathway.

	Course	Year	Details		Study location options
End-to-End Rural Medical Pathway	Bachelor of Biomedical Science (Medical)—La Trobe University.	1	Students build a solid foundation in biomedical sciences through subjects such as, Human Biosciences, Chemistry, and Foundations of Biomedical Sciences. These subjects introduce the basic principles of human biology, chemistry, and the social determinants of health.		Bendigo or Wodonga.
		2	Students delve deeper into biomedical science disciplines, with a focus on, Molecular and Cellular Biology, Biochemistry, Anatomy, Physiology, and Immunology. Students also begin developing research skills, preparing them for evidence-based practice and scientific inquiry.		
		3	Students engage with advanced topics and prepare for clinical settings through subjects like Pathophysiology, Applications of Biotechnology in Pharmacy and Medicine, Clinical Hematology and Biochemistry (the course’s capstone subject), and Infectious Disease Epidemiology. The curriculum emphasizes critical thinking and problem-solving, essential for medical practice.		
	Rural Pathway MD—The University of Melbourne.	1	Students focus on building foundational biomedical science and clinical skills during the first year at the University of Melbourne’s Rural Clinical School in Shepparton. The curriculum integrates body systems, clinical communication, and examination skills while emphasizing early professional identity development. Students engage in problem-based learning to explore the structure and function of body systems and their impact on patient health. Learning is delivered through a blend of webinars, interactive modules, tutorials, and clinical placements in both hospital and community settings. Clinical skills are further supported by peer learning, simulation exercises, and hands-on practice in primary care and hospital environments.		Shepparton.
		2	Students further develop their applied biomedical knowledge, advanced clinical skills, and clinical reasoning. Students rotate through adult medicine, surgery, nesthesia, and emergency medicine, enhancing diagnostic and therapeutic skills. Learning activities include case-based modules, simulation exercises, and clinical skills coaching, which foster critical thinking, communication, and decision-making. The curriculum also emphasizes professional practice, teamwork, ethics, and the physician’s role within the healthcare system.		Shepparton, Wangaratta, or Echuca.
		3	Students build on their clinical exposure across rural and regional health services. Students have the option to maintain continuity by staying embedded in either Shepparton, Echuca, or Wangaratta, or to pursue clinical exposure at alternative sites, including smaller facilities throughout Northeast Victoria. Rural pathway students undertake longitudinal primary care placements, complemented by hospital mini blocks in areas like aged care, child and adolescent health, mental health, and women’s health. These placements develop diagnostic, communication, and management skills while promoting rural generalism as a career path. Students also deepen their understanding of health systems, interprofessional collaboration, and rural care transitions. Learning activities focus on clinical reasoning, risk assessment, and preventative care, with additional professional practice and procedural skills training.	**MD Discovery—Research Scholar subjects:** Students can collaborate on research projects with local clinicians and healthcare providers, addressing issues such as chronic disease management, health disparities, workforce retention, and healthcare gaps.	Shepparton, Wangaratta, Echuca, or Northeast Victoria.
		4	The final year serves as a clinical capstone, consolidating students’ knowledge and preparing them for their transition to internship. The curriculum includes four 4-week clinical placements, where students are fully embedded within healthcare teams across Shepparton, Wangaratta, Ballarat, and Echuca. Through full-time clinical immersion, students actively contribute to patient management and gain independence in clinical decision-making. Teaching activities, including simulation-based training, small group tutorials, online learning, and practical sessions, integrate prior learning with real-world practice. By the end of the year, students will have developed the necessary competencies to transition into internship roles, with a strong commitment to rural healthcare.		Shepparton, Wangaratta, Ballarat, or Echuca.

The University of Melbourne’s Rural Pathway MD (Figure 1 and Table 3) offers a rural-focused medical education, beginning with a first year in Shepparton (Figure 2) that introduces foundational biomedical science and clinical skills through a body systems approach (Table 3). Students engage in blended learning with online modules, small-group tutorials, and clinical placements, focusing on building both scientific knowledge and clinical competencies. The second year immerses students in full-time clinical rotations across Shepparton, Wangaratta, and Echuca, where they further develop their clinical reasoning and diagnostic skills in hospital settings (Table 3). In the third year, students gain extensive rural experience through longitudinal primary care placements and hospital mini blocks, deepening their clinical skills and understanding of rural healthcare systems (Table 3). The final year focuses on preparing students for internship roles through full-time clinical placements across multiple regional locations, integrating prior learning with real-world practice (Table 3). The course intended learning outcomes are presented in Supplementary Table 2.

Building on the diverse experiences in the MD programs, students can tailor their medical education experience by exploring diverse topics beyond the curriculum in the MD Discovery program (Figure 1 and Table 3). Discovery subjects are undertaken in each year of the MD (Table 3 and Supplementary Table 3), progressively building knowledge, skills, and professional experience. The development and implementation of Discovery topics exploring Rural Health and Rural Generalism have been detailed previously (32); however, these topics have not yet been evaluated.

Students spend the entirety of their education in rural communities, where they engage with community-based healthcare teams and patients in hospitals, general practices, and primary care centers. This model of Distributed Medical Education (DME) allows students to engage with rural healthcare systems in a practical and immersive manner, enhancing their understanding of local health needs (15). This is essential for developing the Patient and Medical Practitioner graduate attributes, which emphasize the ability to establish trust with patients, apply sound clinical reasoning, and deliver effective, individualized care in rural settings. Students can decide to remain within the same rural community across multiple years, establishing stronger continuity, which is often the preference (anecdotally).

Rural settings reinforce the Systems of Health Care graduate attribute by building students’ understanding of interdisciplinary roles and developing skills in teamwork, leadership, and systems thinking within smaller, collaborative teams.

### 3.2 Students: Rural background and rural commitment

The student body in the end-to-end rural medical pathway is composed of students from rural areas, who have a strong commitment to working in rural healthcare settings. The small cohort size allows for individualized attention and a supportive learning environment. Each student is actively engaged in their learning, with a focus on self-regulated learning as outlined in the Professional and Leader graduate attribute. Students are encouraged to reflect on their learning progress, identify areas for improvement, and seek feedback to enhance their clinical practice.

### 3.3 Faculty: experienced educators and rural healthcare professionals

The program is delivered by a diverse faculty, which includes academic staff from both La Trobe University and The University of Melbourne, along with practicing rural healthcare professionals who serve as clinical supervisors and mentors. This mix of educators ensures that students receive a blend of theoretical knowledge and practical, real-world experience.

Faculty members are not only experts in their fields but also have a deep understanding of the challenges and rewards of rural practice. Clinical supervisors, who are experienced rural practitioners, provide ongoing mentorship and feedback, allowing students to develop the competencies required for effective rural healthcare delivery. These mentors also help students navigate the complexities of rural medical practice, including resource constraints, patient diversity, and the interdisciplinary nature of rural healthcare teams.

### 3.4 Learning objectives: alignment with AMC standards for assessment and accreditation

The intended learning outcomes of the end-to-end rural medical pathway (Supplementary Tables 1–3) are carefully structured to align with the Standards for Assessment and Accreditation of Primary Medical Programs by the Australian Medical Council (21). Although, revised AMC standards have since come into effect as of the start of 2024 (21), the 2012 AMC standards have been used here as these were the standards being maintained at the time of developing this program. These standards are organized into a thematic framework with four domains:

1. Science and Scholarship: the medical graduate as a scientist and scholar.
2. Clinical Practice: the medical graduate as a practitioner.
3. Health and Society: the medical graduate as a health advocate.
4. Professionalism and Leadership: the medical graduate as a professional and leader.

While the intended learning outcomes for the undergraduate degree place less of an emphasis on the Clinical Practice and Health and Society domains, these learning objectives are designed to guide students through the process of acquiring the knowledge and competencies necessary for progression into the Doctor of Medicine. Mapping all learning objectives from the undergraduate degree and Doctor of Medicine to the AMC standards ensures that each stage of the program builds upon the previous one, with students progressively developing the skills and knowledge required. In addition to the intended learning outcomes, the program also outlines a set of graduate attributes (Table 1), which are collated into a six-domain thematic framework: Self, Knowledge, Patients, Medical Profession, Systems of Healthcare, and Society. The integration of these graduate attributes with the intended learning outcomes provides a comprehensive structure, ensuring that students not only meet the AMC standards but are also equipped to address the multifaceted challenges of rural healthcare practice.

### 3.5 Pedagogical format: integrated learning approaches

Across various phases of the end-to-end pathway the program employs a combination of blended learning, problem-based learning (PBL) (33), simulation, skills workshops, and clinical placements (which include interprofessional education), to ensure students are prepared for the realities of rural medicine.

The program uses a blended learning model, where students access online learning materials and engage in face-to-face sessions. This allows students to learn at their own pace while participating in interactive sessions that deepen their understanding of rural medical issues. PBL is used to foster critical thinking and clinical reasoning skills, encouraging students to engage with real-world medical cases that are directly relevant to rural practice. Through PBL, students learn to synthesize clinical findings, evaluate patient data, and develop management plans. The program includes simulation-based learning, where students practice medical procedures and clinical decision-making in a controlled environment. Skills workshops allow students to refine their abilities in specific areas, such as emergency response, patient communication, and technical procedures.

## 4 Results to date/assessment (processes and tools; data planned or already gathered)

To systematically evaluate the effectiveness of the end-to-end rural pathway in addressing rural workforce shortages, Kirkpatrick’s Four-Level Model of Training Evaluation has been adopted (34). This model provides a structured approach to assessing program outcomes, ranging from student satisfaction, and learning, to behavioral change and long-term workforce impact. By applying this framework, the evaluation ensures a comprehensive analysis of how well the program prepares rural-origin students for medical practice in rural settings, facilitates their transition into the workforce, and contributes to broader healthcare improvements in underserved areas. The first cohort of students will graduate at the end of 2025; hence, evaluation data are incomplete and not ready for reporting. However, the following sections outline the program’s evaluation plan at each level of Kirkpatrick’s model.

### 4.1 Level 1: reaction—student satisfaction and perceptions

To assess initial engagement and satisfaction with the program, longitudinal surveys are conducted to track students’ motivations for rural practice at entry and throughout their training. These surveys capture student perceptions of the curriculum, clinical placements, and overall preparedness for rural medical practice. Additionally, qualitative feedback is collected on their experiences in the end-to-end rural pathway to identify areas for improvement in program delivery and support services.

### 4.2 Level 2: learning—knowledge, skills, and competency development

Student academic performance is systematically tracked to ensure they develop the competencies required for medical practice in rural settings. This includes assessment of coursework, clinical placement performance, and licensing examination results. Progression rates from La Trobe University’s Bachelor of Biomedical Science (Medical) into the University of Melbourne’s Rural Pathway MD are reviewed annually, along with time-to-completion analyses to monitor any delays in training. These data help identify academic or structural barriers that may impact student success.

### 4.3 Level 3: behavior—application of learning in clinical and training environments

The behavior level evaluates how students apply their learning in real-world clinical settings, focusing on their ability to transfer knowledge and skills from the classroom to practice. Experienced rural clinical mentors assess students during placements, observing their integration of clinical knowledge, management of complex cases, teamwork, and communication with diverse patients—core competencies for rural practice.

Students are also required to engage in reflective practice throughout their training. They maintain learning portfolios and participate in self-assessment exercises that encourage them to evaluate their strengths, areas for improvement, and progress toward achieving their learning goals. This process helps to develop self-awareness and a sense of professional responsibility.

Data from ongoing clinical placements, including mentor evaluations and student reflections, will be tracked over time to assess students’ behavioral development and application of learned skills. This will aid in identifying any ways in which the program could better prepare its graduates for rural practice.

### 4.4 Level 4: results—impact on workforce and rural healthcare systems

A key objective of the program is to strengthen the rural medical workforce by increasing the number of graduates practicing in rural areas. Internship positions are available in rural areas, and students are strongly encouraged to seek placements in these regions. To quantify the program’s impact on rural workforce, graduates are tracked longitudinally to assess their practice locations, with a focus on those commencing clinical practice in rural or remote areas (MMM3–7). Particular attention will also be paid to evaluating the place-based impact on workforce, quantifying the proportion of graduates that practice in the same region as the program. Retention rates are monitored yearly up to post-graduation (PGY10) to evaluate long-term workforce distribution. Another key variable is graduates’ medical specialty choice, which will be assessed alongside practice location to provide a more comprehensive understanding of the program’s impact on the workforce and healthcare systems.

Beyond individual graduate outcomes, the program’s broader impact on rural communities and healthcare services is assessed through both qualitative and quantitative measures. This includes evaluating the extent to which the program enhances healthcare service capacity in rural areas, improves access to medical care, and addresses local workforce shortages.

### 4.5 Using evaluation findings for continuous improvement

Findings from these evaluation measures inform curriculum refinements, student support strategies, and targeted incentives to encourage long-term rural practice. The data will also contribute to national policy discussions on rural medical education, providing insights into effective strategies for addressing workforce shortages in underserved areas. By systematically tracking rural practice intentions, training experiences, and workforce outcomes, this evaluation framework ensures the end-to-end rural medical pathway remains responsive to community needs while strengthening the sustainability of the rural medical workforce. Longitudinal tracking of graduates’ practice locations and specialty choices will also help determine whether end-to-end training and rural internship placements contribute to longer-term rural workforce retention.

## 5 Discussion on the practical implications, objectives and lessons learned

### 5.1 Practical implications

The end-to-end rural medical pathway is a significant step toward addressing rural healthcare workforce shortages in Australia. Its innovative, cross-institutional model—developed by La Trobe University and The University of Melbourne—offers an integrated and immersive approach to rural medical education that could be adapted to other underserved regions nationally and internationally. Replicating this model elsewhere would require strategic partnerships tailored to local capacities, ensuring a consistent, rural-focused educational journey. The program’s rural immersion approach embedding students in rural settings from the outset, builds essential competencies in real-world contexts. However, implementation in other areas must consider local infrastructure and ensure access to diverse clinical experiences through partnerships with healthcare providers across primary care, emergency medicine, and public health.

By enabling students to train entirely in rural areas, the program reduces barriers for rural-background students and enhances workforce retention. For other regions, success would depend on the availability of clinical placements, academic support, and local mentors committed to rural practice. Sustainability of the rural workforce is a core objective. For successful adaptation elsewhere, programs must be aligned with the specific health needs of each region whether that’s a demand for generalists or specific specialties. Finally, the program’s social accountability framework grounded in community engagement ensures relevance and responsiveness to rural health priorities. Similar programs should build strong relationships with local stakeholders to co-design curricula, involve community voices in admissions, and foster graduates who are both clinically skilled and committed to health equity.

### 5.2 Objectives

The pathway’s first objective was to provide a seamless rural medical education pathway, via guaranteed entry from the undergraduate degree at two of La Trobe University’s regional campuses into the Rural Pathway MD at The University of Melbourne. This unique feature eliminates the uncertainty that students often face in entering MD programs, particularly those from rural backgrounds. This guaranteed pathway is designed to increase the number of medical professionals working in rural areas, ensuring a stable and reliable pipeline of future healthcare providers. Continued evaluation will determine the full impact of the initiative, but evidence suggests that it is well-positioned to significantly enhance rural healthcare delivery and address workforce shortages across Victoria (4, 6, 7, 9, 35).

The program’s second objective was to immerse students in rural and regional areas from the outset of their studies, utilizing features of DME and place-based learning. This ensures that students are not only academically prepared but also experience the day-to-day realities of rural life and rural practice. This model also reduces the barriers to rural medical education, making it more accessible and appealing to students from rural backgrounds, and it directly addresses the issue of workforce retention in rural healthcare. Once students reach the Doctor of Medicine, this rural immersion also facilitates the development of critical competencies for generalist roles and rural-specific healthcare delivery. This approach aligns with the CBE framework, which emphasizes the mastery of competencies that are particularly relevant to rural medical practice.

### 5.3 Lessons learned

The end-to-end rural medical pathway has provided valuable insights that will inform ongoing program improvement. While the end-to-end rural education model provides unparalleled immersion by providing authentic, real-world learning experiences, a lesson learned is that students must be well-supported during their placements in rural settings. This includes regular contact with faculty, access to mental health resources, and peer networks, to thrive academically and emotionally. Mentorship is also essential. Rural clinicians, often managing heavy workloads, benefit from formal mentorship structures that support both teaching and clinical responsibilities. Similarly, peer support, whether in-person or virtual, helps students stay connected, share experiences, and maintain well-being. Academic support must also be accessible throughout placements. Regular check-ins and flexible access to faculty help students stay aligned with learning outcomes and feel connected despite geographic distance. These supports are critical for developing professional competencies such as teamwork and self-regulated learning, and for fostering long-term commitment to rural practice.

Another key lesson is the need for curriculum flexibility. Rural settings are diverse and dynamic, with varying health needs and resource constraints. Place-based learning, which encourages students to engage directly with rural communities, not only enhances their practical experience but also allows the curriculum to adapt to local health issues and resource constraints. The flexible curriculum is also supported by the Competency-Based Education (CBE) framework, allowing students to progress based on demonstrated competencies. In rural settings, where scope of practice varies, this flexibility ensures students develop essential skills—particularly in generalist roles—aligned with the Medical Practitioner, Health Advocate, and Systems of Health Care graduate attributes. Students may have varying levels of access to resources, healthcare facilities, and learning opportunities, depending on their location. Therefore, the curriculum must provide alternative learning methods, such as online resources, remote mentorship, and virtual learning platforms, to ensure that all students receive the same high-quality education, regardless of where they are placed. This approach not only makes the program accessible to students in diverse rural locations but also allows the program to be more resilient to unexpected challenges.

## 6 Acknowledgment of any conceptual, methodological, environmental, or material constraints

### 6.1 Conceptual constraints

A core assumption is that rural-origin students trained in rural settings will undertake, and remain in, rural practice. While supported by research, this assumption is not without risks, as factors like career opportunities, lifestyle, and family may influence decisions.

The program adapts a national medical curriculum for rural contexts, where healthcare delivery often involves generalized care and resource limitations. While incorporating rural-specific content through DME and place-based learning, challenges remain in balancing standardization with rural realities. Continuous curriculum adaptation is necessary to meet rural healthcare needs while maintaining academic rigor.

### 6.2 Methodological constraints

With placements across diverse rural settings, consistent evaluation is challenging. The variability in healthcare infrastructure and clinical experiences necessitates adapting standardized assessment tools to local contexts. Enhanced coordination across sites is required for reliable, comparable data to inform program improvements.

Tracking graduates’ long-term impact on rural workforce retention is complex, influenced by factors like economic incentives and work-life balance. Effective longitudinal data collection requires sustained engagement with alumni and partnerships with rural health organizations.

### 6.3 Environmental constraints

Rural placements often occur in isolated areas with limited infrastructure, posing challenges for students and faculty. Remote healthcare facilities may offer fewer clinical experiences, and students may struggle with access to academic resources. The program mitigates these issues with subsidized accommodation and remote support services, but further mechanisms are needed for more remote placements.

Collaboration between universities poses challenges in coordinating curricula and resources. Faculty shortages in rural areas also hinder program delivery. The program addresses this by investing in faculty development but maintaining a sustainable faculty supply remains a challenge.

### 6.4 Material constraints

The financial challenges associated with medical education and rural placements may discourage students from rural or disadvantaged backgrounds, particularly as part-time study options are not available. Financial support mechanisms, such as scholarships and subsidized housing, are crucial, but additional funding is needed to ensure accessibility.

Many rural areas lack the necessary technological infrastructure for remote learning and telemedicine. Despite investments in digital resources, ongoing efforts are required to ensure rural placement sites are equipped to meet the program’s needs.

## 7 Conclusion

The Rural Pathway MD represents a transformative approach to addressing rural workforce shortages by embedding students in regional and rural healthcare settings from the outset of their training. By integrating DME principles, eliminating the need for metropolitan placements, and providing structured academic and financial support, the program is designed to maximize rural workforce retention. Institutions aspiring to expand their capacity in DME may consider cross-institutional collaboration, such as outlined here.

As the program continues to evolve, ongoing longitudinal evaluation will be critical in maximizing its full impact on workforce distribution and rural healthcare outcomes. This model has the potential to reshape medical education globally, providing a scalable solution to one of the world’s most persistent healthcare challenges. With sustained policy support and investment, the Rural Pathway MD could serve as a blueprint for training, retaining, and empowering the next generation of rural doctors.

## Data Availability

The original contributions presented in this study are included in this article/Supplementary material, further inquiries can be directed to the corresponding author.
