# Supplementary material for: Support from start to finish—a collaborative primary medical program in rural Victoria, Australia
Source: Front Med (Lausanne). 2025 Jul 21;12:1585017. doi: 10.3389/fmed.2025.1585017 (PMC12319050; doi:10.3389/fmed.2025.1585017)
Supplement: Supplementary file 1 [file Table_1.docx]

**Supplementary Table 1: Bachelor of Biomedical Science (Medical) – Course Intended Learning Outcomes**

| **Learning Outcome** |
| --- |
| - Demonstrate theoretical and technical knowledge in biomedical science that enables scientific rigour in the conduct and evaluation of scientific practice. |
| - Analyse, evaluate and apply scientific information from experiments, reports and peer-reviewed journals. |
| - Communicate biomedical knowledge and ideas to others effectively and professionally in written and oral reports. |
| - Apply biomedical knowledge, technical skills and professional judgement to develop evidence-based solutions to complex biomedical, scientific and technical problems. |
| - Undertake self-directed learning to maintain appropriate professional knowledge and skill in biomedical science. |
| - Work independently and in teams to undertake professional projects and scientific tasks. |
| - Demonstrate knowledge and competencies meeting requirements for progression into a post-graduate medical program. |

**Supplementary Table 2: Rural Pathway MD – Course Intended Learning Outcomes**

| **AMC Competency Domain** | **Learning Outcomes** |
| --- | --- |
| As a Scientist and Scholar | - Applying sound knowledge of normal human structure and function, and the principles of therapeutic interventions to understand the body's response to challenge across the lifespan. - Demonstrating the ability to critically appraise, interpret and apply evidence to answer specific clinical questions, and a commitment to the generation and sharing of new knowledge with patients, the medical community and the general public. |
| As a Medical Practitioner | - Establishing trusting relationships with patients and their caregivers, fully mindful of the patient's individual rights, preferences, social and cultural diversity, to achieve best patient outcomes through shared planning and decision making. - Selecting and performing initial and ongoing patient focussed assessment and appropriate investigations demonstrating adaptations for differing physiological, psychosocial, cultural contexts and illness trajectories. - Using clinical reasoning to synthesise ongoing clinical assessment findings, best medical evidence, population-level data and patient contexts, to prioritise the differential diagnosis and inform the management plan. - Implementing and monitoring tailored management plans that optimise patient outcomes and safety and ensure confidentiality and privacy. |
| As a Health Advocate | - Advocating collaboratively through health promotion, health education and applying the principles of equity and diversity, to address the global, societal, and systemic factors that influence health status. - Working with reciprocity alongside First Nations peoples to collaboratively achieve health advancement. - Employing a structured approach to improve the quality and safety of patient care, through a sound knowledge of the Australian healthcare system, social and environmental accountability and the judicious use of resources. |
| As a Professional and Leader | - Modelling the codes of conduct, including qualities of compassion, honesty, respect, and integrity, along with maintaining physical, emotional and mental health to support personal and professional wellbeing. - Working effectively and respectfully as a leader or co-member of intra- and interprofessional health teams, recognising the contributions of all health care team members to shared learning, to improve patient and population health outcomes. - Engaging as a self-regulated learner, and being aware of one's own capabilities and limitations, along with seeking opportunities for continual improvement of their knowledge and skills to enhance their performance. |

**Supplementary Table 3: Rural Pathway MD – Subject Intended Learning Outcomes**

| **Year** | **Subject** | **AMC Competency Domain** | **Learning Outcome/s** |
| --- | --- | --- | --- |
| 1 | Foundations for Clinical Practice (81.25 credit points) | As a Scientist and Scholar | - Clinician scientist (Knowledge): Use biomedical sciences knowledge to describe normal human development, structure and function across the lifespan, the principles of the body's response to challenge and the foundations for management. - Clinician scientist (Skills): Explain the biomedical processes and mechanisms of common and serious medical conditions, including the clinical features of these conditions and the foundations for management. - Clinician researcher (Knowledge): Describe the key principles of how scientific discovery and evidence are used to support medical practice. - Clinician researcher (Skills): Formulate focused clinical questions, apply the principles of literature searching and identify a range of relevant resources to resolve these questions or identify knowledge gaps. |
|  |  | As a Medical Practitioner | - Partnership with patient (Knowledge): Describe the core skills required to establish relationships with patients and caregivers, being mindful of the patient's individual rights, preferences, social and cultural contexts. - Partnership with patient (Skills): Demonstrate a range of skills to establish rapport and build trust with patients. - Patient assessment (Knowledge): Describe a framework for conducting an initial patient history and physical examination, based on biomedical knowledge and the determinants of health. - Patient assessment (Skills): Perform at a foundation level a patient focused assessment, using an organised framework for history and examination. - Clinical reasoning (Knowledge): Identify the relevant biomedical science knowledge, determinants of health and clinical assessments that can explain patient presentations. - Clinical reasoning (Skills): Derive a list of potential causes for common clinical presentations, integrating knowledge of biomedical sciences and determinants of health with patient assessment findings. - Patient Management (Knowledge): Relate how the biomedical sciences and the determinants of health, inform clinical skills and management plans to support patient outcomes and safety. - Patient Management (Skills): Demonstrate core clinical skills that contribute to patient and practitioner safety. |
|  |  | As a Health Advocate | - Determinants of health (Knowledge): Outline the global, societal and systemic factors that contribute to health status and health inequities for individuals, communities and populations. - Determinants of health (Skills): Demonstrate an understanding of how the determinants of health and patient's own experiences impact on health and health seeking behaviours. - First Nations health (Knowledge): Describe the historic and contemporary factors impacting First Nations peoples, including cultural strength, resilience, and the contribution and value of Indigenous knowledges in advancing health and wellbeing. - First Nations health (Skills): Explain how to apply First Nations health models when working with Aboriginal and/or Torres Strait Islander patients, families and communities, taking a critical approach when reviewing information and resources. - Healthcare systems (Knowledge): Characterise the organisational structure and function of the Australian Healthcare system and recognise what this system offers to individuals, communities and populations. - Healthcare systems (Skills): Identify key health provision structures within the health system that support patient and practitioner safety. |
|  |  | As a Professional and Leader | - Professional practitioner (Knowledge): Describe the core ethical principles that underpin professional and clinical decision making, and the personal qualities that support practitioner well-being necessary for safe practice. - Professional practitioner (Skills): Consistently display professional behaviour encompassing reliability; appropriate interactions with others; willingness to accept and respond to feedback; and personal behaviours in line with relevant codes of conduct and scope of practice. - Collaborative practitioner (Knowledge): Describe how collaborative practices, such as teamwork, interprofessional practice and effective communication, contribute to the quality of healthcare and improved health outcomes for patients. - Collaborative practitioner (Skills): Demonstrate effective teamwork skills by collaboration as members of learning groups. - Reflective practitioner (Knowledge): Describe the foundational skills and attitudes required for self-regulated learning in medicine. - Reflective practitioner (Skills): Demonstrate the foundational skills and attitudes of a self-regulated learner. |
|  | Student Conference 1 (6.25 credit points) | As a Scientist and Scholar | - Describe how a chose relevant student conference learning enhances current understanding of medical practice and/or medical research. |
|  |  | As a Medical Practitioner | - Demonstrate consolidation of clinical skills in their student conference engagement. |
|  |  | As a Health Advocate | - Draw on their learnings at student conference to reflect on their role as a health advocate. |
|  |  | As a Professional and Leader | - Describe how learnings from student conference illustrate the role of a doctor as professional and leader. |
|  | MD Discovery 1: Foundation (12.5 credit points) | As a Scientist and Scholar | - Develop a critical understanding of the relevant theory and knowledge that underpin the nominated area of interest. |
|  |  | As a Medical Practitioner | - Relate their chosen topics to the medical practitioner's role, including patient assessment, clinical reasoning and partnership with patients. |
|  |  | As a Health Advocate | - Draw on their learning to reflect on the role of the of the medical practitioner as a health advocate. |
|  |  | As a Professional and Leader | - Effectively communicate the relationship between Discovery learning and future professional practice. |
| 2 | Principles of Clinical Practice 2 (81.25 credit points) | As a Scientist and Scholar | - Clinician scientist (Knowledge): Apply biomedical sciences knowledge to explain the underlying mechanisms for patient presentations and recommendations for basic management. - Clinician scientist (Skills): Incorporate essential biomedical sciences knowledge in proposing a scientifically supported differential diagnosis, a rationale for the principal diagnosis and a basic management plan for common adult presentations. - Clinician Researcher (Knowledge): Describe the principles of evidence-based practice (EBP) using knowledge of study designs and critical analysis and an understanding of research governance. - Clinician Researcher (Skills): Apply the principles of critical analysis to evaluate the applicability of health-related research to clinical problems. |
|  |  | As a Medical Practitioner | - Partnership with patient (Knowledge): Appreciate and explain the importance of shared decision making with adult patients and their caregivers, being mindful of the patient's individual rights, circumstances and preferences. - Partnership with patient (Skills): Establish relationships with adult patients and their caregivers, being mindful of the patient's individual rights, circumstances and preferences. - Patient assessment (Knowledge): Formulate patient focussed assessments and appropriate initial investigations for adult patients, integrating knowledge of biomedical sciences and determinants of health. - Patient assessment (Skills): Select and perform an appropriate patient focused assessment of an adult, demonstrating adaptations for differing physiological, psychosocial and cultural contexts. - Clinical reasoning (Knowledge): Use clinical reasoning to synthesise assessment findings, population level data and patient context, to prioritise the differential diagnosis and inform a basic management plan for adult patients. - Clinical reasoning (Skills): Synthesise information obtained from interview, examination and preliminary investigations to provide a coherent differential diagnosis and justification for basic management appropriate for the individual. - Patient Management (Knowledge): Explain the key principles of basic management for common and serious conditions and presentations. - Patient Management (Skills): Formulate a basic management plan based on patient assessment findings, ensuring patient safety. |
|  |  | As a Health Advocate | - Determinants of health (Knowledge): Differentiate the global, societal and systemic factors that contribute to health promotion and education within adult patient populations to address health inequities and improve health status. - Determinants of health (Skills): Discuss how to advocate for better health outcomes with individual patients, or patient groups, by application of the principles of equity and diversity within their health experiences. - First Nations health (Knowledge): Examine the impact of health service access and delivery models on the health and well-being of First Nations peoples with recognition of historical and ongoing influences, including government policies. - First Nations health (Skills): Demonstrate the ability to provide culturally safe care with adult First Nations patients and families through developing reflexivity, self-awareness, and person-centred communication skills, as well as utilising First Nations health models in tandem with relevant clinical models. - Healthcare systems (Knowledge): Examine the structures within placement settings that contribute to improvement in quality and safety of adult patient care, using knowledge of the Australian healthcare system, social and environmental accountability, and the judicious use of resources. - Healthcare systems (Skills): Demonstrate a structured approach to improving key elements of patient safety by engaging in quality improvement activities within the hospital setting. |
|  |  | As a Professional and Leader | - Professional practitioner (Knowledge): Analyse the core ethical, legal and moral principles for professional practice in clinical scenarios and experiences, including factors such as personal behaviours, interactions with others, systems and practices that impact on outcomes. - Professional practitioner (Skills): Consistently display professional behaviour encompassing reliability; appropriate interactions with others; willingness to accept and respond to feedback; and personal behaviours in line with relevant codes of conduct and scope of practice. - Collaborative practitioner (Knowledge): Explain how collaborative practices in placement settings enable unique contributions of all health care team members to improve health outcomes of patients. Appraise collaborative practices within healthcare teams, learning groups and the contributions of all team members including themselves. - Collaborative practitioner (Skills): Appraise collaborative practices within healthcare teams, learning groups and the contributions of all team members including themselves. - Reflective practitioner (Knowledge): Explain the skills and attitudes required for self-regulated learning in medicine in a clinical setting. - Reflective practitioner (Skills): Demonstrate the skills and attitudes of a self-regulated learner, being aware of one's own capabilities and limitations in a clinical setting. |
|  | Student Conference 2 (6.25 credit points) | As a Scientist and Scholar | - Explain the key concepts and knowledge bases underpinning a chosen student conference learning that enhance their role as a scientist and scholar. |
|  |  | As a Medical Practitioner | - Demonstrate an extension, or consolidation, of clinical skills through their student conference engagement. |
|  |  | As a Health Advocate | - Discuss how chosen student conference activities enhance their role as a health advocate. |
|  |  | As a Professional and Leader | - Discuss how chosen student conference activities inform their role as a professional and leader. |
|  | MD Discovery 2: Application (12.5 credit points) | As a Scientist and Scholar | - Appraise the key concepts and knowledge bases underpinning their topics. |
|  |  | As a Medical Practitioner | - Apply these concepts to the medical practitioner's role including patient assessment, clinical reasoning and partnership with patients. |
|  |  | As a Health Advocate | - Relate how their chosen topics enhance their role as a health advocate. |
|  |  | As a Professional and Leader | - Demonstrate how their chosen topics inform their professional practice. |
| 3 | Principles of Clinical Practice 3 (81.25 credit points) | As a Scientist and Scholar | - Clinician scientist (Knowledge): Apply biomedical sciences knowledge to explain underlying mechanisms of patient presentations and rationale for management across the age spectrum. - Clinician scientist (Skills): Incorporate essential biomedical sciences knowledge in proposing a scientifically supported differential diagnosis with rationale for the principal diagnosis, management and response to care across the age spectrum. - Clinician Researcher (Knowledge): Integrate knowledge of study design, data analysis, research governance and current clinical practice to identify, interpret and appraise scientific and scholarly evidence. - Clinician Researcher (Skills): Analyse and apply a variety of resources to determine the effectiveness, efficiency and appropriateness of current health care practices in patients across the age spectrum. |
|  |  | As a Medical Practitioner | - Partnership with patient (Knowledge): Appraise the elements required to establish effective relationships with patients and their families and carers, that encompasses shared decision making and tailoring management to their individual preferences and circumstances. - Partnership with patient (Skills): Demonstrate skilful interactions with patients and their families and carers, to ensure shared decision making and tailoring management to their individual preferences and circumstances in all health contexts. - Patient assessment (Knowledge): Tailor frameworks for patient focussed assessment, including investigations, in all health contexts and across the age spectrum, integrating knowledge of biomedical sciences, patient focused care and determinants of health. - Patient assessment (Skills): Assess patients, across all ages, appropriately and accurately, demonstrating adaptations for differing physiological, psychosocial, cultural and care contexts. - Clinical reasoning (Knowledge): Use clinical reasoning to synthesise clinical assessment findings, population level data and patient context to formulate the differential diagnoses and inform the management plan for patients across the age spectrum. - Clinical reasoning (Skills): Synthesise information obtained from clinical assessment findings, collateral information and investigations to provide a coherent differential diagnosis and a rationale for management appropriate for the individual. - Patient Management (Knowledge): Explain the key principles of management for common and serious presentations across the age spectrum including acute and chronic illnesses and incorporating preventative care. - Patient Management (Skills): Implement basic and initial tailored management plans based on patient assessment findings in all healthcare contexts with consideration of collaborative care and ensuring patient safety. |
|  |  | As a Health Advocate | - Determinants of health (Knowledge): Appraise the diverse global, societal and systemic factors that contribute to health status and health inequities, and informs health promotion and education, within all patient populations. - Determinants of health (Skills): Advocate for better health outcomes with individual patients and families in all patient groups, by application of the principles of equity and diversity within their health experiences with an emphasis on continuity of care. - First Nations health (Knowledge): Appraise both community-controlled health organisations and mainstream health services recognising the important role of cultural strengths contributing to health advancement for First Nations' peoples and appraising the influence of biases, judgements, stereotypes, racism and white privilege on First Nations health rights and their healthcare experiences'. - First Nations health (Skills): Perform culturally safe and appropriate clinical assessments with all First Nations' patients and families, and contribute collaboratively to initial management, utilising health models and approaches in tandem with appropriate clinical models, in order to support First Nations health advancement. - Healthcare systems (Knowledge): Evaluate the complexity of coordination and continuity of care within placement settings that contribute to a culture of safety and improvement in the Australian health care system. - Healthcare systems (Skills): Demonstrate a structured approach to improving key elements of patient safety by contributing to quality improvement activities within hospital, speciality health, residential care and community settings. |
|  |  | As a Professional and Leader | - Professional practitioner (Knowledge): Explain the core ethical, legal and moral principles for professional practice to teaching and learning activities, clinical and interprofessional experiences, and appraise factors such as personal behaviours, interactions with others, systems and practices that impact on outcomes for self, patients and colleagues. - Professional practitioner (Skills): Consistently display professional behaviour encompassing reliability; appropriate interactions with others; willingness to accept and respond to feedback; and personal behaviours in line with relevant codes of conduct and scope of practice. - Collaborative practitioner (Knowledge): Appraise the function and purpose of collaborative teams, identifying team members' unique roles in contributing to better patient outcomes in all health care contexts. - Collaborative practitioner (Skills): Participate effectively and respectfully in collaborative healthcare and learning teams to contribute to better patient outcomes whilst sharing their learning with their peers and colleagues from all professions and disciplines. - Reflective practitioner (Knowledge): Articulate the principles and values of reflection for clinical practice and professional development through feedback, awareness of one's limitations and the development responsive learning plans. - Reflective practitioner (Skills): Actively reflect on their practice and seek out formal and informal feedback in order to recognise limits and generate learning plans to continually improve knowledge, skills and attitudes. |
|  | Student Conference 3 (6.25 credit points) | As a Scientist and Scholar | - Critique how conference learning informs the integration of the roles of scientist and scholar to improve the delivery of care to specific patient population. |
|  |  | As a Medical Practitioner | - Select and demonstrate extension of a clinical skill for a self-identified learning gap. |
|  |  | As a Health Advocate | - Illustrate how they could act as a health advocate for a specific patient population/s. |
|  |  | As a Professional and Leader | - Articulate how conference learnings can be integrated into their role as a professional and leader. |
|  | MD Discovery 3: Integration (12.5 credit points) | As a Scientist and Scholar | - Integrate learnings from their topic to extend their knowledge and understanding of clinical scholarship. |
|  |  | As a Medical Practitioner | - Integrate learnings from their topic with core learnings to the medical practitioner's role in their partnership with patient, patient assessment, clinical reasoning and management. |
|  |  | As a Health Advocate | - Appraise how their topic enhances their role as a health advocate. |
|  |  | As a Professional and Leader | - Demonstrate how appropriate learning from their topic is integrated into their professional practice and leadership. |
|  | MD Discovery 3: Research Scholar (12.5 credit points) | As a Scientist and Scholar | - Formulate a research question, to address a gap in the existing research evidence base. - Justify the research design and methodology to answer the formulated research question. - Apply ethical practice principles to the preparation and design of a research project. - Critically analyse and synthesise appropriate scholarly evidence and communicate findings to identify gaps in knowledge and inform research plans. |
|  |  | As a Medical Practitioner | - N/A. |
|  |  | As a Health Advocate | - Discuss how the proposed research plan can inform future practice to advance patient care at a systems level. - Discuss ethical and cultural implications for a specific research proposal, to enable working with reciprocity alongside First Nations peoples to collaboratively achieve health advancement. |
|  |  | As a Professional and Leader | - Discuss ethical and cultural implications for a specific research proposal, referencing the ethical standards for the design, conduct, recording and reporting of clinical research. - Work effectively and respectfully as a co-member of a research team, recognising the contributions of all members to shared learning, to improve patient and population health outcomes. - Engage as a self-regulated learner, to inform professional practice and professional identity as a clinician researcher. |
| 4 | Transition to Practice (43.75 credit points) | As a Scientist and Scholar | - Clinician Scientist (Knowledge): Apply biomedical sciences knowledge to explain the underlying mechanisms of patient presentation, response to care, and rationale for initial and ongoing management. - Clinician Scientist (Skills): Propose a scientifically supported rationale for the entire patient course, including presentation, differential diagnosis, illness trajectory, initial and ongoing management and response to care across the age spectrum. - Clinician Researcher (Skills): Apply the key principles of evidence-based medicine to clinical judgements and collaborative decision making, recognising the individual patient and the heath system and community context. |
|  |  | As a Medical Practitioner | - Partnership with Patient (Knowledge): Appraise the elements required to establish ongoing therapeutic relationships with patients and their families and carers, that encompass shared decision-making and tailoring of treatment goals and management plans to their individual preferences and circumstances. - Partnership with Patient (Skills): Demonstrate skilful interactions with patients, their families and carers, to establish ongoing therapeutic relationships, creating tailored mutually acceptable treatment goals and management plans being mindful of the patient's individual rights, circumstances and preferences in all health contexts. - Patient Assessment (Knowledge): Construct tailored initial and ongoing patient focused assessments, including investigations, in all health contexts integrating knowledge of patient focused care, illness trajectories, biomedical sciences, and determinants of health. - Patient Assessment (Skills): Select and perform appropriate and accurate initial and ongoing patient focused assessments, including gathering of information from relevant sources and demonstrating adaptations for differing physiological, psychosocial, cultural contexts and illness trajectories. - Clinical Reasoning (Knowledge): Use clinical reasoning to develop approaches to synthesise information from initial and ongoing clinical assessment findings, population level data and patient context to formulate the differential diagnoses and inform the management plan for patients across the age spectrum. - Clinical Reasoning (Skills): Synthesise information obtained from initial and ongoing clinical assessment findings, collateral information and investigations, best medical evidence, population level data and patient contexts, to construct and refine a coherent differential diagnosis and inform the management plan for the individual. - Patient Management (Knowledge): Appraise the key principles of initial and ongoing management of common and serious conditions and presentations across the age spectrum including health maintenance, health promotion and disease prevention, management and advance care plans. - Patient Management (Skills): Implement initial tailored management plans based on patient assessment findings, revising these plans for ongoing management through the monitoring of patient responses, in all healthcare contexts with consideration of collaborative care and ensuring patient safety, confidentiality, and privacy. |
|  |  | As a Health Advocate | - Determinants of Health (Knowledge): Evaluate how diverse global, systemic, social and individual factors contribute to health status and health inequities within patient populations. - Determinants of Health (Skills): Advocate for better local and global health outcomes with individual patients, or patient groups, by application of the principles of equity, diversity and sustainability within their health experiences. - First Nations Health (Knowledge): Critically analyse the Australian healthcare system's (including health care services, organisations, governing and accreditation bodies) impact on First Nations peoples' clinical presentations, service delivery and health advancement, and develop strategies for addressing inequities at a systemic level. - First Nations Health (Skills): Apply nuanced understanding of cultural safety and develop strategies for addressing inequities at individual through to systemic levels. - Health Care System (Knowledge): Evaluate the strengths and weaknesses of the coordination and continuity of care that contribute to a culture of safety and improvement in the Australian health care system. - Health Care System (Skills): Employ a structured approach to improving key elements of patient safety by actively engaging in, complying with and contributing to system improvement activities within hospital and community settings. |
|  |  | As a Professional and Leader | - Professional Practitioner (Knowledge): Apply the core ethical, moral and medicolegal principles and processes for professional scenarios and experiences, and critically appraise factors such as personal behaviours, interactions with others, systems and practices that impact on outcomes for self, patients and colleagues. - Professional Practitioner (Skills): Consistently display professional behaviour encompassing reliability, appropriate interactions with others, willingness to accept and respond to feedback, and personal behaviours in line with relevant codes of conduct and scope of practice. - Collaborative Practitioner (Knowledge): Appraise the leadership and teamwork principles that support collaborative intra- and interprofessional practice, recognising the unique contributions of other health care professionals to improve patient and population health outcomes. - Collaborative Practitioner (Skills): Participate effectively and respectfully as a leader and/or member of intra- and interprofessional healthcare and learning teams to contribute to better patient outcomes whilst sharing their learning with their peers and colleagues from all professions and disciplines. - Reflective Practitioner (Knowledge): Articulate the principles and value of reflective practice for clinical practice and professional development, being aware of one's capabilities and how to seek further advice, for performance improvement. - Reflective Practitioner (Skills): Demonstrate engagement in feedback, assessment and continuing professional development opportunities to generate learning plans and continually improve knowledge, skills and attitudes recognising one's own limits and when to seek further advice. |
|  | Student Conference 4 | As a Scientist and Scholar | - Appraise how new learning from novel research presented at conferences might apply to future clinical practice. |
|  |  | As a Medical Practitioner | - Select, demonstrate and explain the importance of extending clinical skills for a self-identified learning gap. |
|  |  | As a Health Advocate | - Evaluate approaches that facilitate their ability to act as a health advocate based. |
|  |  | As a Professional and Leader | - Evaluate approaches that enhance their ability to act as a professional and leader. |
|  | MD Discovery 4: Clinical Scholar (50 credit points) | As a Scientist and Scholar | - Apply ethical practice principles in the conduct of research or project work. - Critically interpret, synthesise and communicate findings from a range of scientific and scholarly literature. - Justify the choice of a research design or project work and methodology to address a research question or clinical need. - Utilise strategies for keeping up to date and practising medicine in a specific context in line with the latest evidence. |
|  |  | As a Medical Practitioner | - Perform clinical tasks (within the student's scope of practice) in their chosen field demonstrating patient assessment clinical reasoning and management in partnership with patients. |
|  |  | As a Health Advocate | - Justify how their clinical engagement in a chosen field and scholarly output supports future practice to advance patient care at a systems level. - Critique the role of the clinician scholar in advocating for and collaborating with First Nations patients and community to achieve health advancement and develop strategies for addressing inequities at an individual community and societal levels. |
|  |  | As a Professional and Leader | - Engage as a self-regulated learner to inform professional practice, leadership, and professional identity as a clinician scholar. - Work effectively and respectfully as a co-member and leader of a clinical team, recognising the contributions of all members to shared learning, to improve patient and populations health outcomes. |
|  | MD Discovery 4: Research Scholar | As a Scientist and Scholar | - Conduct research using the methodology outlined in a project plan. - Analyse data using appropriate methods described in a project plan. - Apply ethical practice principles to the conduct of a research project. - Communicate research findings and conclusions to the medical community and the general public. |
|  |  | As a Medical Practitioner | - N/A. |
|  |  | As a Health Advocate | - Examine how the findings of the research project could inform future practice to advance patient care. - Critique the role of the clinician researcher in advocating for, and collaborating with, First Nations patients and community to achieve health advancement and develop strategies for addressing inequities at an individual, community and societal level. |
|  |  | As a Professional and Leader | - Demonstrate how engaging in research contributes to professional practice and develops professional identity as a researcher. - Work effectively and respectfully as a co-member of a research team, recognising the contributions of all members to shared learning, to improve patient and population health outcomes. - Engage as a self-regulated learner to conduct supervised research. |
